# Immunoglobulin M profile of viral and atypical pathogens among children with community acquired lower respiratory tract infections in Luzhou, China

**DOI:** 10.1186/s12887-019-1649-6

**Published:** 2019-08-13

**Authors:** Ai Chen, Liyao Song, Zhi Chen, Xiaomei Luo, Qing Jiang, Zhan Yang, Liangcai Hu, Jinhua He, Lifang Zhou, Hai Yu

**Affiliations:** 1 Department of Pediatrics, The Affiliated Hospital of Southwest Medical University , No. 25, Taiping Street, Jiangyang District, Luzhou, 646000 Sichuan Province China; 2 Standardized training nurse from 2019, The Affiliated Hospital of Southwest Medical University, Luzhou, 646000 Sichuan Province China; 3 Chongqing Jiulongpo District Maternal and Child Health Care Family Planning Service Center, Chongqing, 400050 China; 4 Traditional Chinese medicine hospital of Jiangbei district, Chongqing, 400020 China; 5 Undergraduate students in Grade 2012, clinical college of Southwest Medical University, Luzhou, 646000 Sichuan China

**Keywords:** Children, Community-acquired lower respiratory tract infections, Respiratory pathogens, IgM antibodies

## Abstract

**Background:**

Community-acquired lower respiratory tract infections (CA-LRTIs) are the primary cause of hospitalization among children globally. A better understanding of the role of atypical pathogen infections in native conditions is essential to improve clinical management and preventive measures. The main objective of this study was to detect the presence of 7 respiratory viruses and 2 atypical pathogens among hospitalized infants and children with community-acquired lower respiratory tract infections in Luzhou via an IgM test.

**Methods:**

Overall, 6623 cases of local hospitalized children with 9 pathogen-IgM results from 1st July 2013 to 31st Dec 2016 were included; multidimensional analysis was performed.

**Results:**

1) Out of 19,467 hospitalized children with lower respiratory tract infections, 6623 samples were collected, for a submission ratio of 33.96% (6623 /19467). Of the total 6623 serum samples tested, 5784 IgM stains were positive, for a ratio of 87.33% (5784 /6623). *Mycoplasma pneumoniae* (MP) was the dominant pathogen (2548 /6623, 38.47%), with *influenza B* (INFB) (1606 /6623, 24.25%), *Legionella pneumophila serogroup 1* (LP1) (485 /6623, 7.32%) and *parainfluenza 1, 2 and 3*(PIVs) (416 /6623, 6.28%) ranking second, third and fourth, respectively.

2) The distribution of various pathogen-IgM by age group was significantly different *(χ*^2^ = 455.039, *P* < 0.05).

3) Some pathogens were found to be associated with a certain age of children and seasons statistically.

**Conclusions:**

The dominant positive IgM in the area was MP, followed by INFB, either of which prefers to infect children between 2 years and 5 years in autumn. The presence of atypical pathogens should not be underestimated clinically as they were common infections in the respiratory tract of children in the hospital.

## Background

CA-LRTIs are the primary cause of hospitalization among children globally [1]. Recent estimates suggest that nearly 120 million new cases of community-acquired pneumonia (CAP) occur each year, with almost 1 million deaths among children aged < 5 years [2]. In 2016, CAP killed an estimated 880 000 children, accounting for the death of approximately 2400 children per day [3]. However, our knowledge about the CA-LRTIs is still lacking.

Bacterial pathogens remain a major cause of CA-LRTIs in children, leading to continuous morbidity and mortality, particularly in developing areas. He et al. [4] reported that *S. aureus*, *E. coli*, and *K. pneumonia* were the common bacterial isolates recovered from children with CA-LRTIs during 2011–2015 in Dongguan. However, an increasing number of studies have reflected that many childhood CA-LRTIs are caused by atypical pathogens. It is reported that the new strain of influenza A virus subtype H1N1 afflicted at least 394,133 people in Asia in 2009, which frightened many people [5].

An updated surveillance on influenza activity in the U.S.A. during 30th September 2018 -2nd February, 2019 from the CDC demonstrated H1N1-pdm09 viruses predominated in most areas of the country, while *influenza A virus subtype H3N2* (H3N2) viruses were prevalent in the southeastern United States [6].

In addition, MP,*adenovirus* (ADV) and other viruses are significantly implicated in LRTIs at present. Nationwide surveillance data originating from Stockholm, Sweden, indicated that influenza virus, *metapneumovirus*, and *respiratory syncytial virus* (RSV) were detected in 60% of their enrolled cases in 3 years [7], with similar results in Australia, showing that in developed countries, 7 to 48% of young children with CAP have RSV detected in respiratory specimens [8]. Additionally, studies from developing countries (Vietnam [9] and India [10]) illustrated that RSV was the most predominant pathogen detected locally. Obviously, a better understanding of the role of atypical pathogen infections in native conditions is essential to improve clinical management and preventive measures.

The reason why the prevalence of each pathogen varies from region to region may mostly be due to seasonal and geographic factors, as well as the heterogeneous status of the population. Luzhou is a city located in Sichuan Province, a region in southwest China. It is a metropolitan area with a population greater than 5 million residents. In addition, it borders Yunnan and Guizhou Provinces and the Chongqing district and is the only geographic junction of these four areas. Therefore, exploring the etiology of CA-LRTIs in Luzhou is significant for the health of the associated children. Much research demonstrates that an indirect immune-fluorescence technique for IgM detection is a reasonably sensitive, highly specific, and cost-effective approach for the identification of viral or atypical bacterial pathogens [11]. We adapted an IgM kit that can simultaneously diagnose 9 pathogens of the respiratory tract for infectious diseases, including MP, LP1, *chlamydia pneumoniae* (CP), ADV, *Coxiella burnetii* (COX), RSV, *influenza A* (INFA), INFB, and PIVs. The study aimed to elucidate the etiologic spectrum of atypical pathogens by their immunoglobulin M and indirectly investigate the distribution of 9 pathogens of CA-LRTIs in children to assess whether there is an association between age or season and the etiological organism. All the data were extracted by engineers from the hospital information system (HIS), which is an element of health informatics and filtered by the inclusion criteria.

## Methods

### Overview

Overall, 6623 cases of local hospitalized children with 9 pathogen-IgM results from 1st July 2013 to 31st December 2016 were included; multidimensional analysis was performed retrospectively.

### Study population

The Department of Pediatrics of the Affiliated Hospital of Southwest Medical University is a tertiary care center with over 300 beds, including the Department of Pediatric Emergency Unit, Department of Neonatal Intensive Care Unit, Department of Pediatric Intensive Care Unit, and several vital departments of common pediatric internal medicine. The number of daily outpatient visits is approximately 400. This retrospective study was conducted with patients less than 14 years old displaying symptoms of CA-LRTIs. Enrolled participants met three inclusion criteria as follows: 1) presence of one or more respiratory symptoms, including wheezing, cough, dyspnea, phlegm production, pleuritic pain, or/and fever; 2) physical examination that illustrated abnormal traits, such as tachypnea, tri-concavity signs, rales or rhonchi on chest auscultation; and 3) evidence of pneumonia/bronchitis or other inflammations by radiography, such as a chest X-ray or computed tomography (CT) scan. The results were interpreted by attending radiologists separately as showing pulmonary opacity, such as consolidation, interstitial, nodules, and atelectasis. The exclusion criteria were hospital-acquired LRTIs, i.e., pneumonia that developed 72 h after hospitalization or within 7 days of discharge.

### Weather data abstraction

Luzhou is situated in the southeast region of Sichuan Province, at longitude 105° 08′ 41″E ~ 106° 28′E and latitude 27° 39′ N ~ 29° 20′N. Since the Yangtze River flows through the whole area from west to east, it is characterized as a river valley with mild and humid weather. The annual temperatures fluctuate from 2.6 °C to 39 °C. Weather temperature data were collected from a China weather search website (http://tianqi.2345.com/wea_history/57602.htm).

Since the total data collection ended on 31st December 2016, only three complete season circles were included. We chose the matched data of 6533 cases within the three complete season circles (from 1st September 2013 to 31st August 2016) to explore whether climatological factors influence the atypical pathogens.

### Serology

Atypical infectious agents refer to those pathogens uncommon to cause the usual disease. Nine pathogen-linked immunosorbent assays were performed for immunoglobulin M antibodies. Two milliliters of patient serum samples was collected and sent to the laboratory. Serum IgM antibodies against LP1, MP, COX, CP, ADV, RSV, INFA, IFNB and PIVs were detected using available commercial ELISA-based kits following the manufacturer’s instructions (Vircell tech Inc. Granada, Spain. China agency code: 13 M224). Briefly, 25 μl of Vercelli ELISA sorbent and 75 μl of serum dilution were added to the corresponding wells according to the kit instructions. The interpretative criteria were consistent with the recommendations of the manufacturer. The complete process was manipulated by technicians in the laboratory of the Affiliated Hospital of Southwest Medical University, whose microbiological laboratory quality assurance was in accordance with the Clinical & Laboratory Standards Institute (CLSI) guidelines.

### Statistical analysis

Statistical analysis was performed using the statistical software SPSS 21.0 (IBM Corp, Armonk, NY). A Spearman correlation test was used to observe the association between age variables. A chi-square test was used to determine the significance of differences in incidence between the seasons, and a *p*-value < 0.05 was considered statistically significant.

## Results

### Patients’ characteristics

In total, we analyzed 6639 samples collected from LRTI patients (age 0–14 years) during a 3.5 year period. The most frequent clinical diagnoses were pneumonia (81.80%), bronchiolitis (1.52%), and bronchitis (16.67%).

### The positive percentage of 9 pathogens

As far as CA-LRTIs are concerned, from 1st July 2013 to 31st Dec 2016, a total of 19,467 hospitalized pediatric patients met the inclusion criteria. The mean age of these patients was 1.73 years (standard deviation: 2.46 years; range 0–14 years). Of the 19,467 patients with CA-LRTIs, the majority were younger than 1-year old (46.27%, 9007/19467). Toddlers between 1 and 2 years old contributed 15.89% (3094/19467), and 2- to 5-year-old children represented 17.23% (3355/19467), higher than the 5- to 10-year-old group (6.50%,1266/19467) and teenagers (1.91%, 371/19467).

**Fig. 1:**
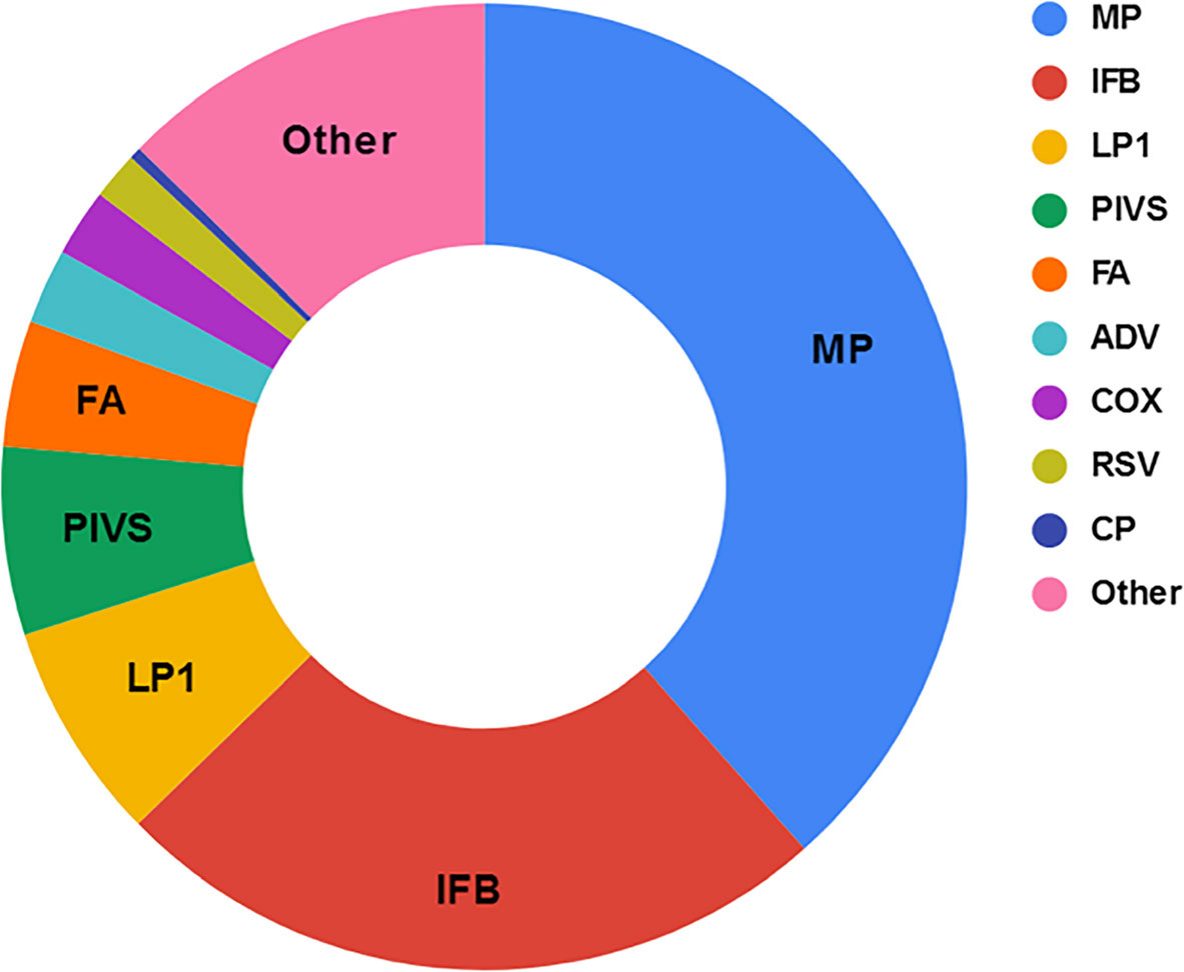
IgM of pathogens distribution from 6623 children with CA-LRTIs. Among 34.02% (6623 /19467) submission samples, 5784 stains IgM were identified, the ratio was 87.33%(5784 /6623). The most frequent pathogen was MP, (2548 /6623, 38.47%), followed by INFB (1606 /6623, 24.25%), LP1 (485 /6623, 7.32%), PIVs (416 /6623, 6.28%) and INFA (281 /6623, 4.24%).The four tails were ADV (166 /6623, 2.51%), COX (150 /6623, 2.26%), RSV (106 /6623, 1.60%) and CP (26 /6623, 0.39%)

Nevertheless, only 6623 children were enrolled with the agreement of their guardians; unfortunately, we did not extract gender information. The mean age of these patients was 1.74 years (standard deviation: 2.44 years; range 0–14 years). Among 34.02% (6623 /19467) of the submission samples, 5784 stains IgM were identified, for a ratio of 87.33% (5784 /6623). The most frequent pathogen was MP (2548 /6623, 38.47%), followed by INFB (1606 /6623, 24.25%), LP1 (485 /6623, 7.32%), PIVs (416 /6623, 6.28%) and INFA (281 /6623, 4.24%). The four least frequent pathogens were ADV (166 /6623, 2.51%), COX (150 /6623, 2.26%), RSV (106 /6623, 1.60%) and CP (26 /6623, 0.39%) (Fig. 1).

### Pathogen IgM distribution in different age groups

A total of 5784 cases of atypical respiratory pathogens with positive IgM antibodies were divided into 5 groups according to age (Table 1), and the susceptibility of each group to atypical respiratory pathogens was versatile.

**Table 1:** Distributions of IgM of 9 pathogens by age (n, %)

Age (year)	MP	LP1	ADV	INFA	INFB	PIVs	RSV	CP	COX	Co-infections
< 1 (1151)	18.52 (472)	7.85 (38)	29.52 (49)	41.99 (118)	14.01 (225)	32.93 (137)	66.03 (70)	11.54 (3)	26.00 (39)	15.93 (277)
1–2 (1407)	23.37 (672)	21.81 (106)	34.34 (57)	23.13 (65)	23.29 (374)	17.54 (73)	18.87 (20)	7.69 (2)	25.33 (38)	23.81 (414)
2–5 (2202)	37.72 (961)	43.91 (213)	25.3 (42)	26.69 (75)	43.77 (703)	33.17 (138)	10.38 (11)	30.77 (8)	34.00 (51)	41.52 (722)
5–10 (817)	13.93 (355)	20.21 (98)	9.64 (16)	5.69 (16)	15.57 (250)	12.74 (53)	3.77 (4)	34.62 (9)	10.67 (16)	15.30 (266)
> 10 (207)	3.45 (88)	6.19 (30)	1.20 (2)	2.49 (7)	3.36 (54)	3.61 (15)	0.94 (1)	15.38 (4)	4.00 (6)	3.45 (60)
Total = 5784	100 (2548)	100 (485)	100 (166)	100 (281)	100 (1606)	100 (416)	100 (106)	100 (26)	100 (150)	100 (1739)

Group definition:

< 1 year: infants less than1 year

1–2 year: ≥1 year and < 2 year

2–5 year: ≥2 year and < 5 year

5-10 year:≥5 year and < 10 year

≥10 year: children older than 10 year

Spring includes Mar, Apr, and May; summer includes Jun, Jul, and Aug; autumn includes Sep, Oct and Nov; winter includes Jan, Feb and Dec

To be detailed, IgM of INFA and RSV were more commonly isolated in infants less than 1 year old, CP and ADV mainly attack 1–2-year-olds. For those children between 2 to 5 yeas old, MP, INFB, and LP1 are the common strains in their respiratory tracts. PIVs usually infected groups younger than 1 year and ages between 2 to 5 years. Totally, the portions which children’s age from 2 to 5 years old were more common population with atypical infectious agents causing CA-LRTIs. Meanwhile, co-infection should be paid attention that 277, 414, 722, 266, 60 cases matching their age growing groups involved two or more agents when detected (Table 1).

**Fig. 2:**
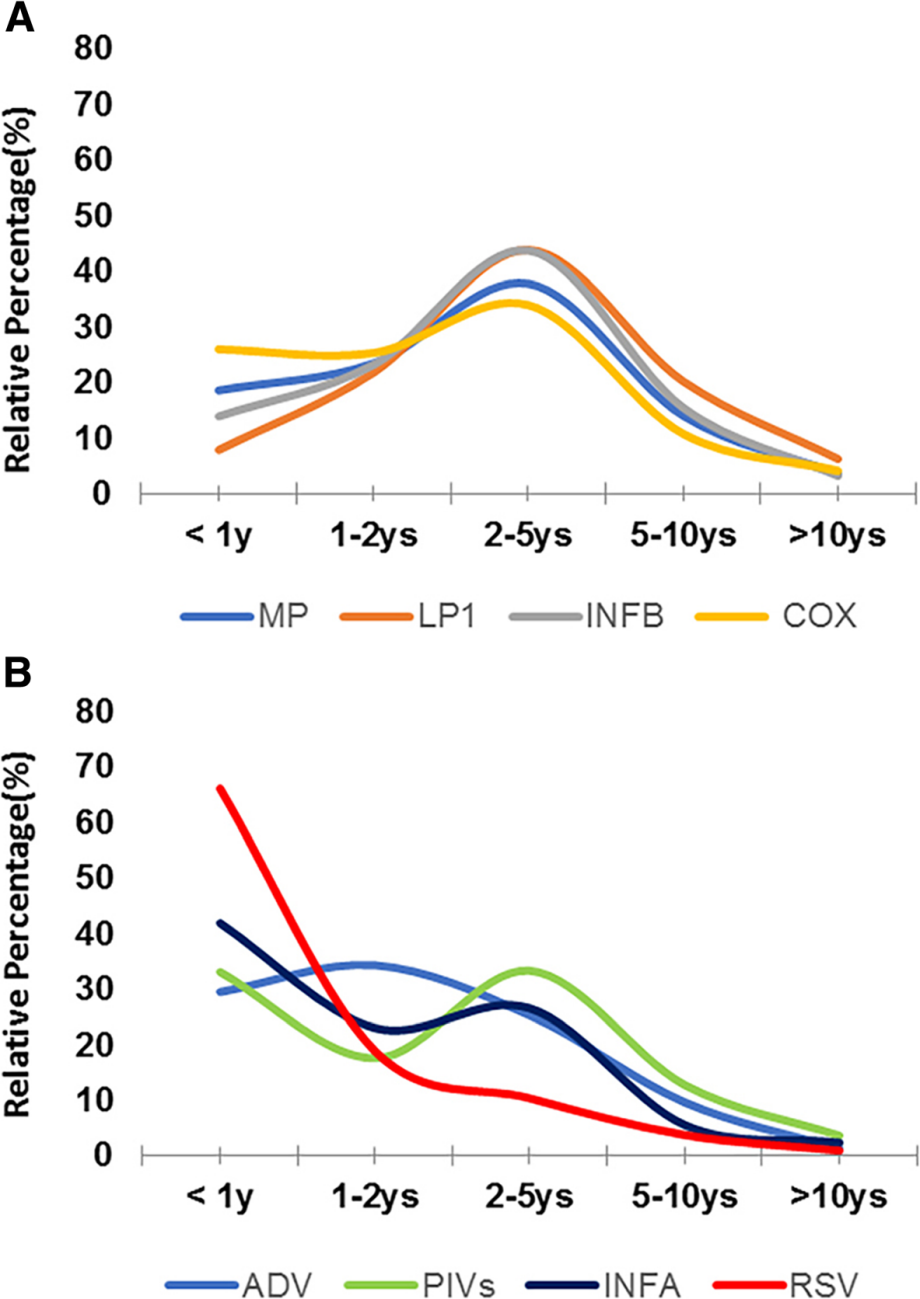
Distributions of IgM of 9 pathogens in ages by percentage. MP, LP1, INFB and COX appear the similar curve which suggested they peaked in 2–5 years group and then the susceptibility of MP and IFB was significantly declined after 5 years of age (**a**). Another pattern demonstrated as (**a)**, RSV almost lured in < 1 year babies arriving 70%, with INFA, PIVs and ADV closely followed. They were less and less popular with the age increasing, as there is a direct or zigzag slope shown in (**b**)

Age distribution: By the percentage of each pathogen (Table 1), we can easily determine their susceptibility tendency in different age groups. It is shown in that MP, LP1, INFB, and COX exhibit similar curves, which suggested that they peaked in the 2 to 5-year-old group and that the susceptibility of MP and INFB significantly declined after 5 years of age (Fig. 2a). As shown in Fig. 2a, RSV was found in many babies younger than 1 year, reaching a prevalence of 70%, with INFA, PIVs and ADV following closely behind. These infections were less prevalent with increasing age, as indicated by the director zigzag slope shown in Fig. 2b.

### Monthly distributions of pathogen IgM

**Fig. 3:**
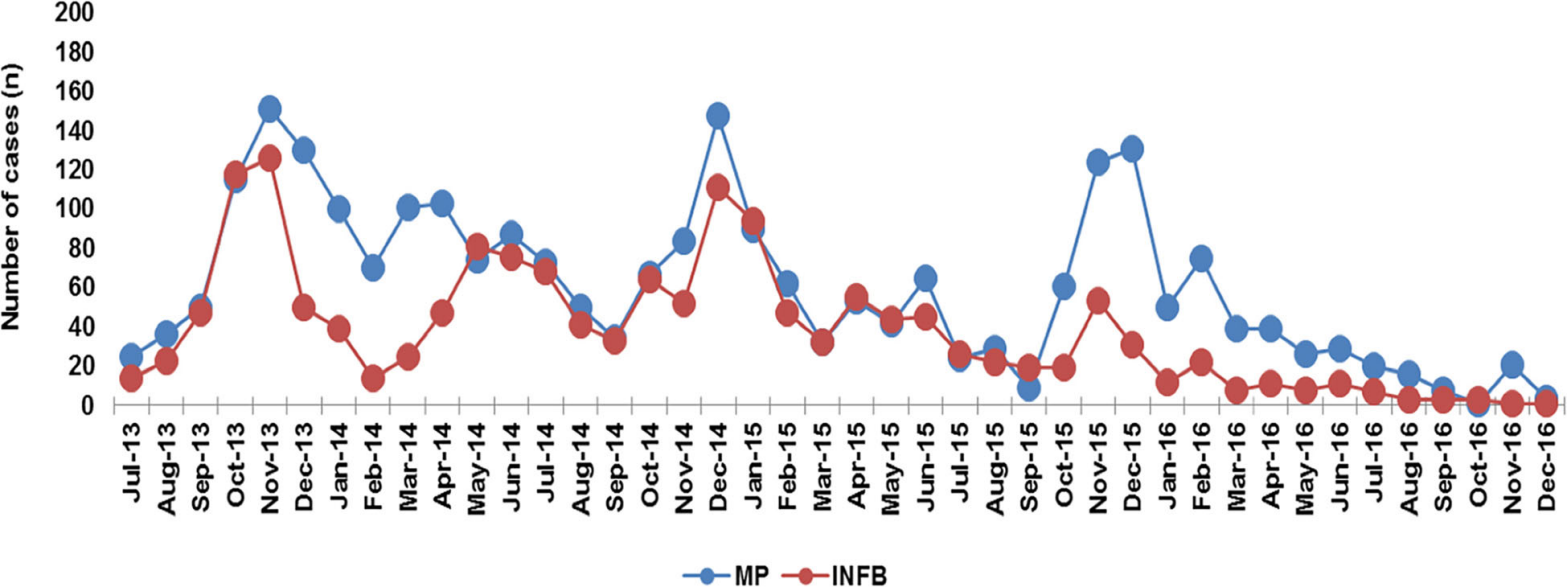
Monthly Distribution of MP and INFB Ig M. A fluctuated distribution of infections with MP as well as INFB were observed across the year and there was a relative increase from November to January annually. The peaks of MP and INFB were always high through the winter

**Fig. 4:**
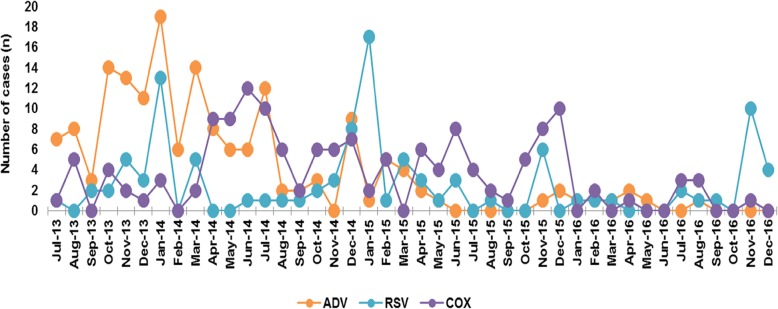
Monthly Distribution of ADV, RSV, COX IgM. There was a relative increase from November to January annually shown in IgM distribution of RSV. There was an obvious decreased tendency with ADV distribution from 2013 to 2016. There was an obvious decreased distribution with ADV infectious number from almost 20 in 2013 to less than 5 in 2015–2016.Since relative less COX -IgM cases in each year, there were unobvious regular chores illustrated in COX -IgM distribution

**Fig. 5:**
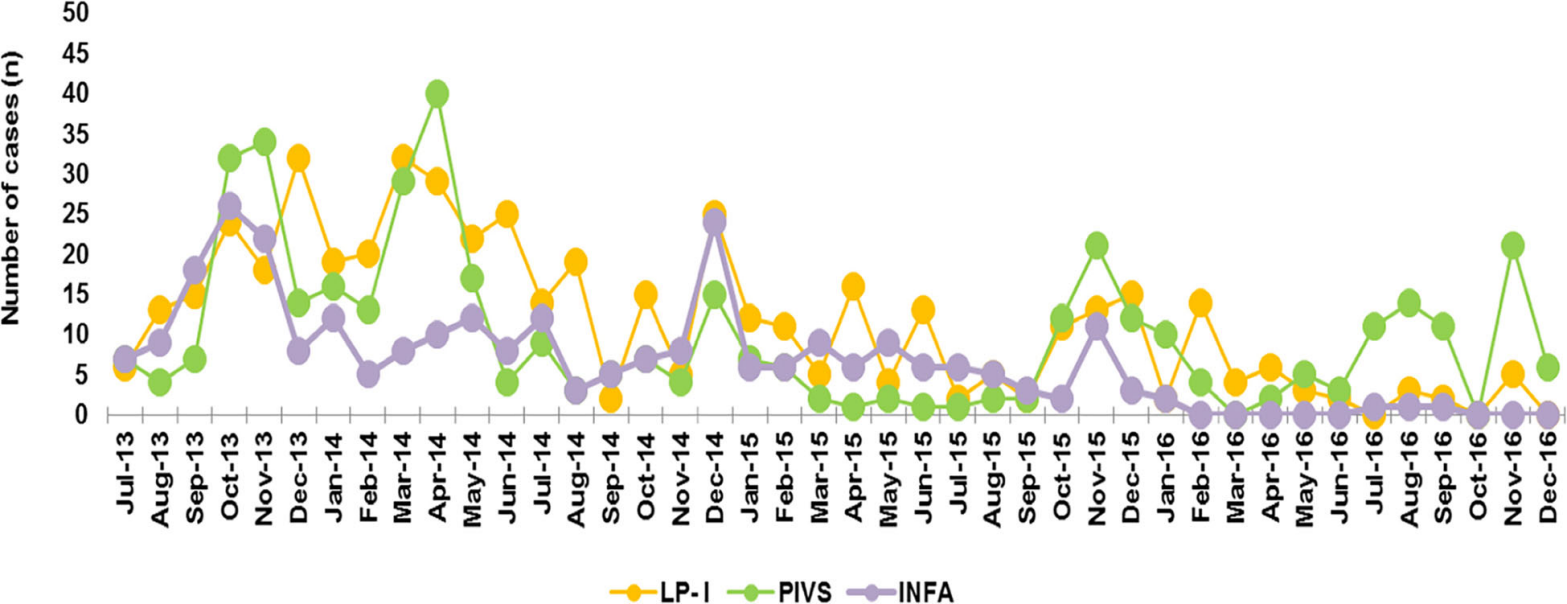
Monthly Distribution of LP1, PIVs, INFA Ig M. Besides a summit appeared in April of year 2014, PIVS IgM distribution in winter (November) were sensitive annually. INFA seems silent expressed during year 2016 after its positive shown in year 2013–2015. LP-1 infection peaked on December of year 2013–2015

The monthly distribution of the CA-LRTIs cases was analyzed according to their etiologies during the study period. A fluctuating distribution of infections with MP as well as INFB was observed throughout the year, and there was a relative increase from November to January annually; in other words, peaks of MP and INFB were always high throughout the winter (Fig. 3). A parallel trend was also observed in RSV (Fig. 4). There was an obviously decreased distribution of ADV infections from almost 20 cases in 2013 to less than 5 cases in 2015–2016. Since relatively fewer COX-IgM cases were observed in each year, there were unobvious regular patterns illustrated in the COX-IgM distribution (Fig. 4). Concerning the PIVS IgM distribution, other than the peak occurring in April 2014, it was likely that October and November were the seasons for children to get PIVS (Fig. 5). INFA appeared to be silently expressed during the year 2016 after it was positive in 2013–2015 (Fig. 5). There were no trends detected in LP-1 infection, except that it peaked in December of 2013–2015 (Fig. 5).

### Seasonal distribution of CA-LRTIs and the pathogen IgM

To analyze the seasonal positive rate of CA-LRTIs, we define the season as spring (March–May), summer (June–August), autumn (September–November), and winter (December–February) (Table 2). Due to the total data collection ended on 31st December 2016, only three complete season cycles included. We chose the matched data 6533 cases within the three complete season circles (From 1st Sep 2013 to 31st Aug 2016) to analyze whether climatological factors can influence atypical pathogens concurrence. According to the glossary of American Mathematical Society, as an index of climate, the cumulative lowest and highest temperature were calculated from the daily minimum and maximum temperatures in a certain period, which widely used to evaluate the influence of metabolomic changes to pathogens [12]. Besides, considering we need to calculate the *p*-value, a specific metabolomic number is better than an average temperature (mean ± SD) for performing the calculation. Then we finally chose the cumulative temperature as our data for reference to local meteorology. According to variance normality and homogeneity assumptions, the chi-square test was used to determine the significance of differences in positive rate between the seasons (Table 2).

**Table 2:** Seasonal distribution of the Pathogens IgM (n, %)

Year		The cumulative lowest temperature (°C)	The cumulative highest temperature (°C)	Cases	MP	INFB	COX	RSV	ADV	LP1	PIVs	INFA	CP
2013	autumn	1419	1972	1018	316 (31.04)	291 (28.58)	6 (0.58)	9 (0.88)	30 (2.94)	57 (5.59)	73 (7.17)	66 (6.48)	2 (0.19)
	winter	532	993	715	300 (41.95)	103 (14.40)	4 (0.55)	16 (2.23)	36 (5.03)	71 (9.93)	43 (6.01)	25 (3.49)	1 (0.13)
2014	spring	1410	2065	830	278 (33.49)	153 (18.43)	20 (2.40)	5 (0.60)	28 (3.37)	83 (10.00)	86 (10.36)	30 (3.61)	1 (0.12)
	summer	2073	2741	649	210 (32.35)	185 (28.50)	28 (4.31)	3 (0.46)	20 (3.08)	58 (8.93)	16 (2.46)	23 (3.54)	2 (0.30)
	autumn	1492	1971	517	185 (35.78)	149 (28.82)	14 (2.70)	6 (1.16)	5 (0.96)	22 (4.25)	16 (3.09)	20 (3.86)	2 (0.38)
	winter	634	1109	811	300 (36.99)	252 (31.07)	14 (1.72)	26 (3.20)	15 (1.84)	48 (5.91)	28 (3.45)	36 (4.43)	2 (0.24)
2015	spring	1482	2204	417	127 (30.45)	131 (31.41)	10 (2.39)	9 (2.15)	7 (1.67)	25 (5.99)	5 (1.19)	24 (5.75)	3 (0.71)
	summer	2092	2759	328	118 (35.97)	93 (28.35)	14 (4.26)	4 (1.21)	0 (0.00)	20 (6.09)	4 (1.21)	17 (5.18)	3 (0.91)
	autumn	1542	2013	471	194 (41.18)	91 (19.32)	14 (2.97)	6 (1.27)	1 (0.21)	26 (5.52)	35 (7.43)	16 (3.39)	0 (0.00)
	winter	573	1014	439	256 (58.31)	65 (14.80)	12 (2.73)	2 (0.45)	4 (0.91)	31 (7.06)	26 (5.92)	5 (1.13)	2 (0.45)
2016	spring	1382	2074	191	104 (54.45)	27 (14.13)	1 (0.52)	1 (0.52)	4 (2.09)	13 (6.80)	7 (3.66)	0 (0.00)	2 (1.04)
	summer	2125	2886	147	65 (44.21)	21 (14.28)	6 (4.08)	3 (2.04)	1 (0.68)	5 (3.40)	28 (19.04)	2 (1.36)	1 (0.68)
			Chi-square test		157.123	158.175	49.978	39.741	59.342	40.585	147.312	42.449	a
			*p*		< 0.01	< 0.01	< 0.01	< 0.01	< 0.01	< 0.01	< 0.01	< 0.01	b

Spring includes Mar, Apr, and May; summer includes Jun, Jul, and Aug; autumn includes Sep, Oct and Nov; winter includes Jan, Feb and Dec

The seasonal distribution of CA-LRTIs in patients showed the highest incidence of CA-LRTIs in autumn (*n* = 2006; 30.70%), followed by winter (*n* = 1965; 30.07%) and spring (*n* = 1438; 22.01%), and the lowest incidence was recorded in summer (*n* = 1124; 17.20%). According to the chi-square analysis, the incidence of eight pathogens other than CP in different seasons was statistically significant (*P* < 0.01) (Table 2).

**Fig. 6:**
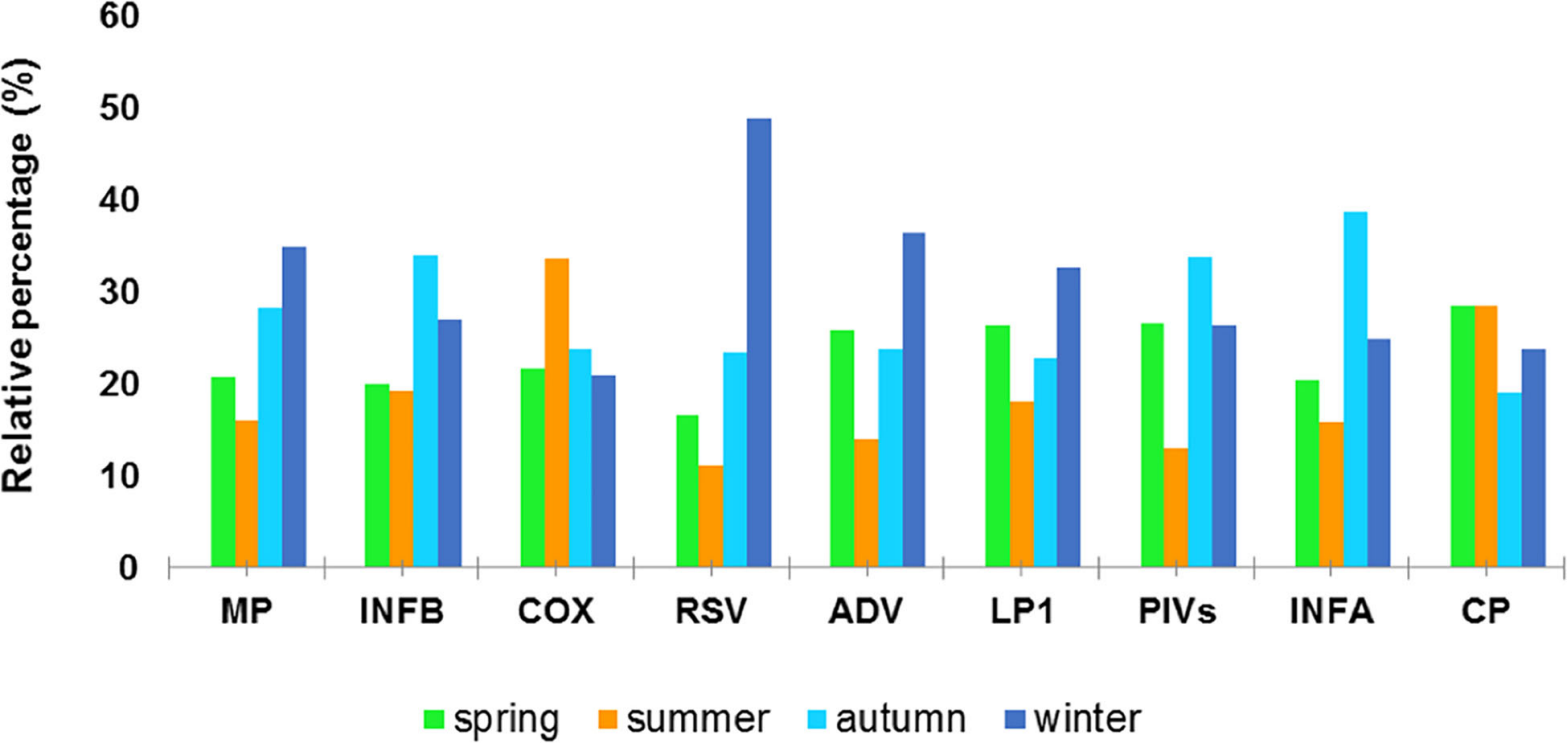
Seasonal Distributions of IgM of 9 pathogens by percentage. There are seasonal differences in the susceptibility of CA-LRTIs children to 9 respiratory pathogens; the peak positive rate of MP, LP1, RSV and ADV are more common in winter; while the peak positive rate of INFA, INFB, and PIVs is more obvious in autumn. The COX and CP always activated in summer

There were seasonal differences in the susceptibility of CA-LRTIs children to 9 respiratory pathogens; the peak positive rates of MP, LP1, RSV and ADV were more common in winter; while the peak of the positive rate of INFA, INFB, and PIVs was more obvious in autumn. The COX and CP were always active in summer (Fig. 6).

As Table 3 shown, with the influence of seasonal factors, according to Spearman correlation analysis, the positive rate of MP and INFB (*P* < 0.01), RSV (*P* = 0.04), ADV (*P* < 0.01), LP1 (*P* < 0.01), PIVs and INFA (*P* = 0.01) were correlated (*P* < 0.05); There was a strong correlation among ADV, INFA and INFB. Especially, the correlation coefficient for INFB with INFA is 0.909. (*R* = 0.909, *P* < 0.01).

**Table 3:** Correlation between 9 respiratory pathogen IgM antibodies and cumulative temperature of seasons(R, P)

	MP	INFB	COX	RSV	ADV	LP1	PIVs	INFA	CP
The lowest temperature	−0.6 (0.03)	−0.14 (0.64)	0.33 (0.28)	−0.27 (0.38)	−0.54 (0.06)	−0.49 (0.1)	−0.33 (0.29)	−0.3 (0.33)	0.07 (0.8)
The highest temperature	−0.72 (0.00)	− 0.25 (0.41)	0.14 (0.65)	− 0.46 (0.12)	− 0.47 (0.11)	− 0.49 (0.1)	− 0.44 (0.14)	− 0.36 (0.24)	0.21 (0.49)
MP		0.71 (0.00)	0.14 (0.64)	0.59 (0.04)	0.78 (0.00)	0.87 (0.00)	0.68 (0.01)	0.80 (0.00)	−0.19 (0.55)
INFB	0.71 (0.00)		0.44 (0.14)	0.61 (0.03)	0.71 (0.00)	0.66 (0.01)	0.03 (0.34)	0.90 (0.00)	0.21 (0.51)
COX	0.14 (0.64)	0.44 (0.14)		0.00 (0.98)	−0.01 (0.95)	0.35 (0.26)	0.00 (0.99)	0.24 (0.44)	0.01 (0.95)
RSV	0.59 (0.04)	0.61 (0.03)	0.00 (0.98)		0.52 (0.07)	0.41 (0.17)	0.36 (0.24)	0.79 (0.00)	−0.03 (0.90)
ADV	0.78 (0.00)	0.71 (0.00)	−0.01 (0.95)	0.52 (0.07)		0.82 (0.00)	0.55 (0.06)	0.80 (0.00)	−0.12 (0.69)
LP1	0.87 (0.00)	0.66 (0.01)	0.35 (0.26)	0.41 (0.17)	0.82 (0.00)		0.64 (0.02)	0.74 (0.00)	−0.27 (0.38)
PIVs	0.68 (0.01)	0.30 (0.34)	0.00 (0.99)	0.36 (0.24)	0.55 (0.06)	0.64 (0.02)		0.48 (0.11)	−0.76 (0.00)
INFA	0.80 (0.00)	0.90 (0.00)	0.24 (0.44)	0.79 (0.00)	0.80 (0.00)	0.74 (0.00)	0.48 (0.11)		0.07 (0.82)
CP	−0.19 (0.55)	0.21 (0.51)	0.01 (0.95)	−0.03 (0.9)	−0.12 (0.69)	− 0.27 (0.38)	−0.76 (0.00)	0.07 (0.82)	

Note: Cumulative temperature and MP IgM antibody positive rate were correlated: MP was negatively correlated with cumulative maximum temperature (Table 2) (*P* < 0.05); correlation coefficient was − 0. 722.Which means that every 1 °C decrease in the cumulative minimum temperature, the positive number of MP IgM antibodies increased by 0.103 (the regression equation: MP case = 347.687–0.103* cumulative minimum temperature)

As shown in Table 3, the cumulative temperature and MP IgM antibody positive rate were correlated: MP was negatively correlated with cumulative maximum temperature (*P* < 0.05); the correlation coefficient was − 0. 722. This means that for every 1 °C decrease in the cumulative minimum temperature, the positive number of MP IgM antibodies increased by 10.3%.

## Discussion

LRIs are defined as radiologically or clinician-confirmed pneumonia, bronchiolitis and other inflammation in the lower respiratory tract infections based on WHO. A study summarized the burden of LRIs in 195 countries in 2015 provides an analysis that under-5 LRI mortality occurred in 1048 children per 100 000 and estimated that LRIs were the fifth-leading cause of death globally [2]. At the same time, according to a systematic analysis focusing on cause-specific child mortality in China between 1996 and 2015 [13], pneumonia still contributed to a higher proportion of deaths in the western region of China than in the eastern and central regions and remains the main cause of death in rural areas, although there has been dramatic improvement in the under-5 LRI mortality rate. Measures to protect, prevent, and treat LRIs are highlighted in the Global Action Plan [14]. Renewing efforts to control and prevent LRIs depend on the degree to which we understand the disease. Some solutions to prevent LRI deaths do not require major advances in technology. The emergence and precise diagnosis with essential pathogen identification have been much more successful in reducing the deterioration caused by LRIs. Typically, the pathogens causing children’s ALRIs are still dominated by bacteria, and with the application of broad-spectrum antibiotics, the hospitalization duration of ALRI caused by common bacterial infections has been gradually shortened [15]. In contrast, viruses and atypical respiratory pathogens are highly overlooked due to the non-specific clinical manifestations of ALRI, such as wheezing, coughing or hypoxia, and there is overlap among these syndromes. Therefore, the timely identification of viruses and atypical respiratory pathogens is beneficial for differentiating viral, bacterial or other ALRIs in children.

Viruses are responsible for a large proportion of LRTIs in children, and rapid identification of viral infections can help control their transmission. Additionally, studies using currently approved rapid tests or direct fluorescent antibody testing have already demonstrated improvements in clinical practice [16–18]. In this study, indirect immunofluorescence was used to rapidly detect 9 respiratory pathogens considered to be the usual suspects for LRTI that have been sought previously [19]:RSV, INFA, INFB, PIVs and ADV combined with MP, CP, and COX. The technique is suitable for rapid clinical screening, which can easily be carried out with the desired sensitivity in an ordinary laboratory with a basic fluorescence microscope and kit.

Our studies have demonstrated that in 19,467 cases with ALRI, the number of IgM antibody samples was 34.02% (6623 /19467), which is still far behind the ratio observed in developed countries or the eastern region of China, suggesting that pathogen tracking awareness needs to be improved in doctors and parents. However, among the 6623 specimens delivered, 5784 cases (87.33%, 5784 /6623) were positive, suggesting the sensitivity the detected method had. Among them, the MP positive rate was the highest, reaching 44.05% (2548 /5784), far more than the rate (17.40%, 133 /764) of children tested positive for MP by PCR or serology in Denmark [20]. Actually, the much lower rate in Denmark may be due to the different method to detect pathogens. Many results from various regions have demonstrated that MP usually attacks older children [21]. However, our study showed that almost 82.61% of MP-infected children were less than 5 years old (Table 1, Fig. 1). The age-related subgroups indicated that 2–5-year-olds contributed 37.72%, followed by 1–2-year-olds, accounting for 23.37%, and infants younger than 1 year represented up to 18.52%, similar but slightly lower data of South Africa [22].

Tian et al. [23] recruited pneumonia patients from the department of pediatrics in Hangzhou and found the MP detection rate was significantly higher in summer to autumn than in winter to spring. A fluctuating distribution of infections with MP as well as INFB was observed throughout the year, and there was a relative increase from November to January annually (Fig. 2). The chi-square analysis showed that the incidence of MP was more common in winter (*P* < 0.01), which suggested that cold temperature may be the risk factor for the local children to get MP infection. After incorporating the influence of seasonal factors, there was a relatively close coefficient incidence of MP and INFB (*P* < 0.01), RSV(*P* = 0.04), and ADV (*P* < 0.01). For every 1 °C decrease in the cumulative minimum temperature, the number of positive MP IgM antibodies in infected children increased by 10.3%.

Of the samples tested in our study, 38.88% were positive for viruses, which is less than the 81.6% of cases positive for viruses collected from Mexican children younger than 5 years old with CAP in a national multicenter study. RSV is a common cause of childhood ALRI and a major cause of hospital admissions in young children worldwide, resulting in a substantial burden on health-care services. Approximately 45% of hospital admissions and in-hospital deaths due to RSV-ALRI occur in children younger than 6 months [21] and are estimated to be responsible for up to 22% of severe LRTIs in children under 5 years of age. For example, 23.7% children had RSV infection in the Mexican study, while parainfluenza virus (types 1–4) was found in 5.5%, influenza virus (types A and B) in 3.6%, and ADV in 2.2% [21]. In Bulgaria, during the 2014/15 and 2015/16 winter seasons, viral respiratory pathogens were detected in 429 (70%) out of 610 patients examined, and RSV was the most frequently identified virus (26%) [24]. Although our data on RSV found that only 1.6% (106/6623) of the samples was positive, consistent with mainstream research, it was found to mostly infect infants younger than 1 year old (66.03%, 70/106) in winter (Table 1).

Rather than RSV, we found that IgM of INFB ranked 2nd at 27.77% of the pathogens examined, while RSV was only 1.6%. The specific distribution is possibly due to the varied region or enthics [25] since data from other studies showed that 18.7% tested positive for influenza virus out of 666,493 specimen in the USA [26].while H3N2 viruses predominated in the southeastern United States, only small numbers of < 3% INFB were reported [6]. In addition,37.7% hospitalized children in Argentina had influenza, among them, 91.4% had INFA, and 8.6% had INFB [27]. For the seasonal availability and age of children analyzed in our study, INFA preferred to infect children < 1 year, and INFB infected children 2–5 years, and both were more active in autumn (Tables 1 and 2; Fig. 2).

Another frequent infection in autumn and spring was PIVs, which contributed to 6.28% (416/6623) of cases. Children < 1 year and 2–5 years were more highly infected. The same distribution was found in Hebei, China, between March 2014 and February 2015; the positive rate of PIV-3 from 5150 children with ALRTI was 439 cases/8.52%, with the highest in May (21.38%) and the lowest in November [28]. In contrast, PIVs peaked in autumn, and the low was in summer in our city. ME et al. [29] found that PIV1 and PIV3 were most common (31 and 32.5% of total PIV positive samples, respectively), with distribution being similar in children and adults. It is easily spread from parents to children through close contact and classically linked to mild respiratory symptoms such as wheezing (1.77%). Therefore, educating parents to prevent the spread of PIVs by kissing is necessary.

Furthermore, several of the other pathogens found were LP1, ADV, COX, and CP. *Legionella* are ubiquitous in the environment and are particularly prevalent in man-made habitats, such as water distribution systems, possibly leading to an outbreak in the community [30]. *Legionella* is the causative agent of *Legionnaires*’ disease (LD), which involves severe pneumonia that is transmitted through inhalation of contaminated aerosols. The most common species to cause disease is *L. pneumophila*, which has 16 serogroups, but the majority of human disease is caused by *L. pneumophila serogroup* (sg) 1 [31]. In Nanjing, China, the positive percentages of LP1 are found in August and September. A total of 485 samples in our research were positive, the main proportion was found in toddlers 2–5 years old, and winter was the popular season. Another assumption is that *L. pneumophila* easily attacks immune-deficient children, such as those with tuberculosis, tumors, and HIV. It is essential to detect the source of infection promptly by comparing clinical and environmental isolates so that decontamination measures can be implemented to prevent further cases [32]. From 2013 to 2016 in Luzhou, pediatric ADV infection dramatically decreased in our monitored data (Figs. 2 and 4). A study during five consecutive seasons (2011–2016) in Belgium confirmed that children under the age of 6 were most likely to catch an acute respiratory infection caused by ADV [33], with higher rates in winter.

Q fever is a worldwide zoonosis caused by COX, but with few studies conducted to date, very little is known about the epidemiology of rickettsioses in China. A 25-year nationwide study in Israeli children illustrated that almost all cases were treated with a long-term antibiotic regimen [34]. However, the average duration of hospitalization of 150 IgM positive cases in our study was only 7.54 days. Together with only 26 CP positive samples, we found that COX and CP were always activated in summer (Figs. 2 and 6). Our study also revealed that 1739 cases were coinfections, representing a high positive rate (26.25%, 1739/6623) of the specimens (Table 4). However, the exact coexistence pattern was not analyzed due to the complexity of possible dual, triple or multiple coinfections.

**Table 4:** Seasonal distribution of the Pathogens IgM overall (n, %)

Season	MP	INFB	COX	ADV	LP1	PIVs	INFA	CP
Spring	509 (20.75)	311 (19.92)	31 (21.68)	39 (25.83)	121 (26.36)	98 (26.70)	54 (20.45)	6 (28.57)
Summer	393 (16.02)	299 (19.15)	48 (33.57)	21 (13.91)	83 (18.08)	48 (13.08)	42 (15.91)	6 (28.57)
Autumn	695 (28.33)	531 (34.02)	34 (23.78)	36 (23.84)	105 (22.88)	124 (33.79)	102 (38.64)	4 (19.05)
Winter	856 (34.90)	420 (26.91)	30 (20.98)	55 (36.42)	150 (32.68)	97 (26.43)	66 (25.00)	5 (23.81)

Spring includes Mar, Apr, and May; summer includes Jun, Jul, and Aug; autumn includes Sep, Oct and Nov; winter includes Jan, Feb and Dec

## Conclusions

This is the first study to investigate the etiological profile of respiratory atypical pathogens in children hospitalized with CA-LRTIs in Luzhou, which is located in Sichuan Province in the southwest region of mainland China. We provide an overview of the prevalence and seasonality of 9 respiratory pathogens causing CA-LRTIs in different age groups over 3 consecutive respiratory seasons, which strongly suggested that in addition to bacterial infections, pediatric physicians should pay attention to the atypical pathogens. As observed in our results, the IgM of MP was the most prevalent, followed by INFB and LP1 sequentially. In addition, some pathogens were found to be statistically associated with age and season. These data may have implications for the management of patients, which will assist in developing better strategies for therapy and prevention by halting the spread of pathogens in susceptible age groups during peak seasons.

### Limitations

There were some limitations in our study. First, although the IgM test was reasonably sensitive and specific for the detection of pathogens, the results should be verified by specific DNA PCR methods if possible. However, it was unperformable due to economic and staff reasons. Second, as a retrospective study, the samples from healthy groups as control were unavailable because of the ethnic principles. Third, the exact pattern of coinfections was not listed out systematically due to the complexation of the data. Finally, clinical manifestation and radiography data should have been collected and analyzed accordingly to make the elaboration more meaningful.

## Data Availability

The datasets used for the current study are available from the corresponding author on reasonable request.

## Abbreviations

ADV: Adenovirus
CA-LRTIs: Community-acquired lower respiratory tract infections
CAP: Community-acquired pneumonia
COX: Coxiella burnetii
CP: Chlamydophila pneumoniae
E coli: *Escherichia coli*
H1N1: Influenza A virus subtype H1N1
H3N2: Influenza A virus subtype H3N2
IgM: Immunoglobulin M
INFA: Influenza A
INFB: Influenza B
K. pneumonia: *Klebsiella pneumoniae*
LP1: *Legionella pneumophila* serogroup 1
MP: Mycoplasma pneumoniae
PIVs: Human parainfluenza 1, 2 and 3
RSV: Respiratory syncytial virus
*S. aureus*: *Staphylococcus aureus*

## References

[1] Lafond KE, Nair H, Rasooly MH (2016). Global role and burden of influenza in pediatric respiratory hospitalizations, 1982-2012: a systematic analysis. PLoS Med.

[2] GBD 2015 LRI Collaborators. Estimates of the global, regional, and national morbidity, mortality, and aetiologies of lower respiratory tract infections in 195 countries: a systematic analysis for the Global Burden of Disease Study 2015. Lancet Infect Dis. 2017;17(11):1133–61. https://www.thelancet.com/journals/laninf/article/PIIS1473-3099(17)30396-1/fulltext. 10.1016/S1473-3099(17)30396-1 PMC5666185 28843578

[3] AKC L, AHC W, Hon KL (2018). Community-acquired pneumonia in children. Recent Patents Inflamm Allergy Drug Discov.

[4] He X, Xie M, Li S (2017). Antimicrobial resistance in bacterial pathogens among hospitalized children with community acquired lower respiratory tract infections in Dongguan, China (2011-2016). BMC Infect Dis.

[5] Sparke M, Anguelov D (2012). H1N1, globalization and the epidemiology of inequality. Health Place.

[6] Blanton L, Dugan VG, Abd EAI (2019). Update: influenza activity - United States, September 30, 2018-February 2, 2019. MMWR Morb Mortal Wkly Rep.

[7] Scheltema NM, Gentile A, Lucion F (2017). Global respiratory syncytial virus-associated mortality in young children (RSV GOLD): a retrospective case series. Lancet Glob Health.

[8] Shi T, McAllister DA, O'Brien KL (2017). Global, regional, and national disease burden estimates of acute lower respiratory infections due to respiratory syncytial virus in young children in 2015: a systematic review and modelling study. Lancet.

[9] HKL N, Nguyen SV, Nguyen AP (2017). Surveillance of severe acute respiratory infection (SARI) for hospitalized patients in northern Vietnam, 2011-2014. Jpn J Infect Dis.

[10] Sonawane Anuja A., Shastri Jayanthi, Bavdekar Sandeep B. (2019). Respiratory Pathogens in Infants Diagnosed with Acute Lower Respiratory Tract Infection in a Tertiary Care Hospital of Western India Using Multiplex Real Time PCR. The Indian Journal of Pediatrics.

[11] Aguilera-Alonso David, López Ruiz Rocío, Centeno Rubiano Jose, Morell García Marta, Valero García Isabel, Ocete Mochón María Dolores, Montesinos Sanchis Elena (2019). Características clínicas y epidemiológicas de las neumonías adquiridas en la comunidad por Mycoplasma pneumoniae en una población española, 2010-2015. Anales de Pediatría.

[12] Lai YH (2018). The climatic factors affecting dengue fever outbreaks in southern Taiwan: an application of symbolic data analysis. Biomed Eng Online.

[13] He C, Liu L, Chu Y (2017). National and subnational all-cause and cause-specific child mortality in China, 1996-2015: a systematic analysis with implications for the sustainable development goals. Lancet Glob Health.

[14] Qazi S, Aboubaker S, MacLean R (2015). Ending preventable child deaths from pneumonia and diarrhoea by 2025. Development of the integrated global action plan for the prevention and control of pneumonia and Diarrhoea. Arch Dis Child.

[15] López-Alcalde J, Rodriguez-Barrientos R, Redondo-Sánchez J (2018). Short-course versus long-course therapy of the same antibiotic for community-acquired pneumonia in adolescent and adult outpatients. Cochrane Database Syst Rev.

[16] González LA, Vázquez Y, Mora JE (2018). Evaluation of monoclonal antibodies that detect conserved proteins from respiratory syncytial virus, Metapneumovirus and adenovirus in human samples. J Virol Methods.

[17] Dabaja MF, Greco G, Villari S (2018). The first serological study of Q fever in humans in Lebanon. Vector Borne Zoonotic Dis.

[18] Yıldırım D, Özdoğru SD, Şeflek B (2017). Detection of influenza virus infections by molecular and immunofluorescence methods. Mikrobiyol Bul.

[19] Lu YY, Luo R, Fu Z (2017). Pathogen distribution and bacterial resistance in children with severe community-acquired pneumonia. Zhongguo Dang Dai Er Ke Za Zhi.

[20] Søndergaard MJ, Friis MB, Hansen DS (2018). Clinical manifestations in infants and children with mycoplasma pneumoniae infection. PLoS One.

[21] Watkins K, Sridhar D (2018). Pneumonia: a global cause without champions. Lancet.

[22] Carrim M, Wolter N, Benitez AJ (2018). Epidemiology and molecular identification and characterization of mycoplasma pneumoniae, South Africa, 2012-2015. Emerg Infect Dis.

[23] Tian DD, Jiang R, Chen XJ (2017). Meteorological factors on the incidence of MP and RSV pneumonia in children. PLoS One.

[24] Korsun N, Angelova S, Tzotcheva I (2017). Prevalence and genetic characterisation of respiratory syncytial viruses circulating in Bulgaria during the 2014/15 and 2015/16 winter seasons. Pathog Glob Health.

[25] Schuster JE, Williams JV (2018). Emerging respiratory viruses in children. Infect Dis Clin N Am.

[26] Garten R, Blanton L, AIA E (2018). Update: influenza activity in the United States during the 2017-18 season and composition of the 2018-19 influenza vaccine. MMWR Morb Mortal Wkly Rep.

[27] Gentile A, Lucion MF, Del VJM (2018). Influenza virus: 16 years’ experience of clinical epidemiologic patterns and associated infection factors in hospitalized children in Argentina. PLoS One.

[28] Li QH, Gao WJ, Li JY (2016). Detection of respiratory viruses in children with acute lower respiratory tract infection: an analysis of 5,150 children. Zhongguo Dang Dai Er Ke Za Zhi.

[29] Álvarez-Argüelles ME, Rojo-Alba S, Pérez MZ (2018). New clinical and seasonal evidence of infections by human Parainfluenzavirus. Eur J Clin Microbiol Infect Dis.

[30] Shivaji T, Sousa PC, San-Bento A (2014). A large community outbreak of legionnaires disease in Vila Franca de Xira, Portugal, October to November 2014. Euro Surveill.

[31] Diederen BM (2008). Legionella spp. and Legionnaires’ disease. J Inf Secur.

[32] Wolter N, Carrim M, Cohen C (2016). Legionnaires’ disease in South Africa, 2012-2014. Emerg Infect Dis.

[33] Ramaekers K, Keyaerts E, Rector A (2017). Prevalence and seasonality of six respiratory viruses during five consecutive epidemic seasons in Belgium. J Clin Virol.

[34] Sachs N, Atiya-Nasagi Y, Beth-Din A (2018). Chronic Q fever infections in Israeli children: a 25-year Nationwide study. Pediatr Infect Dis J.

